# Supplementary material for: Biodistribution and safety of a single rAAV3B-AAT vector for silencing and replacement of alpha-1 antitrypsin in Cynomolgus macaques
Source: Mol Ther Methods Clin Dev. 2024 Jan 30;32(1):101200. doi: 10.1016/j.omtm.2024.101200 (PMC10914479; doi:10.1016/j.omtm.2024.101200)
Supplement: Document S1. Figures S1–S4 and Tables S1 and S2 [file mmc1.pdf]

OMTM, Volume *32*

Supplemental information

Biodistribution and safety of a single rAAV3B-AAT vector for silencing and replacement of alpha-1 antitrypsin in *Cynomolgus macaques*

Meghan Blackwood, Alisha M. Gruntman, Qiushi Tang, Debora Pires-Ferreira, Darcy Reil, Oleksandr Kondratov, Damien Marsic, Sergei Zolotukhin, Gwladys Gernoux, Allison M. Keeler, Christian Mueller, and Terence R. Flotte

Supplemental Figures

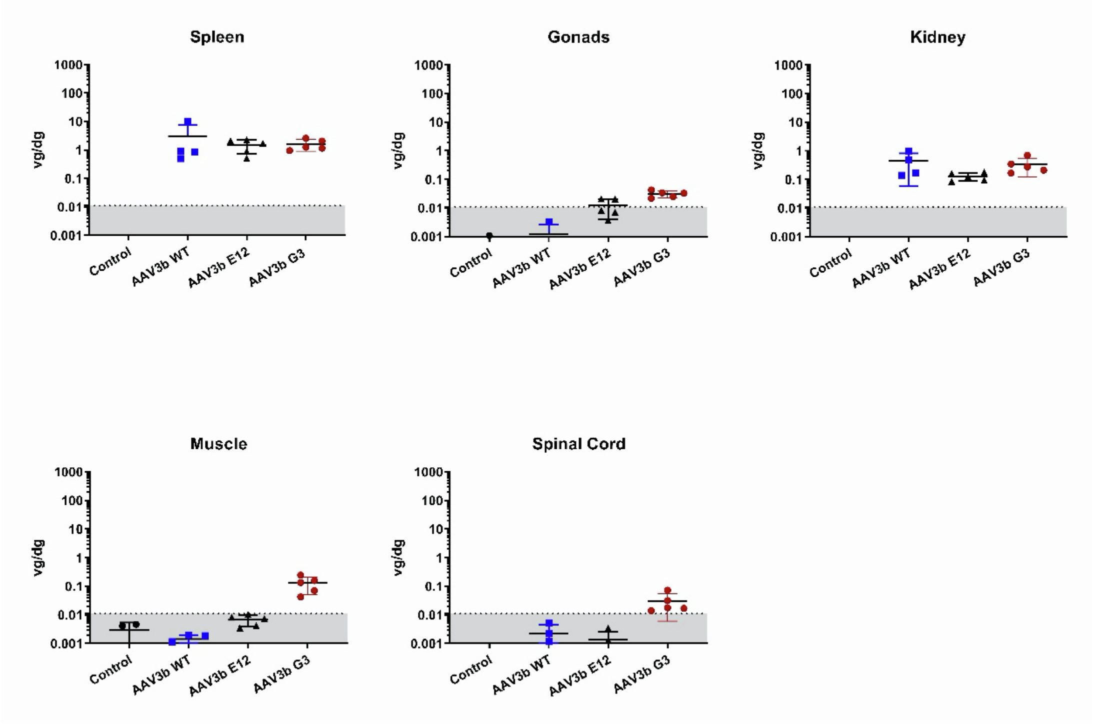

Figure S1 Biodistribution of the rAAV genome in several tissues showed no significant differences among the groups.

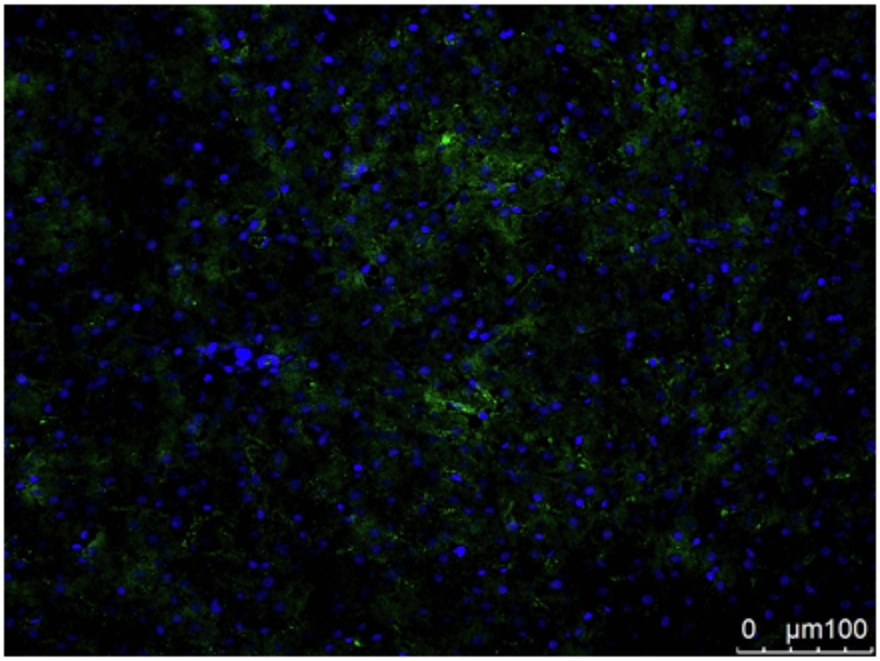

Figure S2 c-MYC staining of liver tissue from uninjected NHP showing minimal background fluorescence. Green: c-MYC; blue: DAPI.

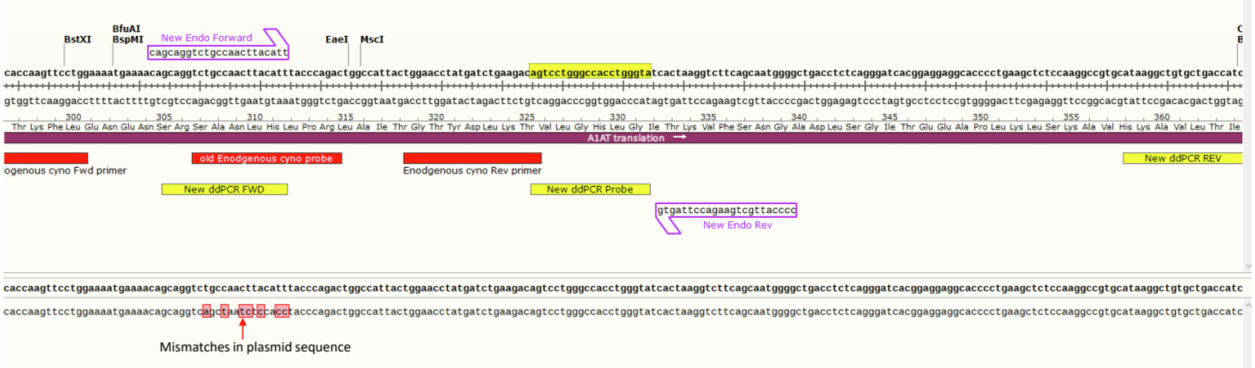

Figure S3 Design of primers for quantifying endogenous AAT gene levels and silent mutations in the gene intended to cause mismatches with the miRNA.

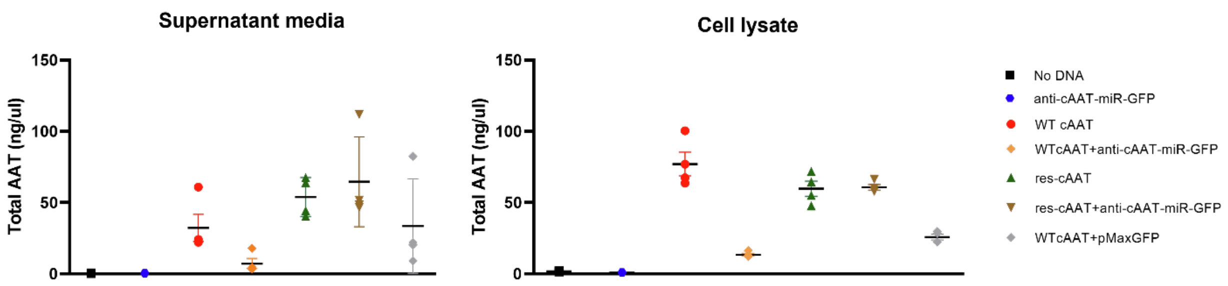

Figure S4 We designed experiments to confirm that constructs based on the cynomolgus alpha-1 antitrypsin (cAAT) sequence were effective for expression of wild-type cAAT (WT cAAT), silencing cAAT (anti-cAAT-miR) and expressing an allele of cAAT that is resist to that silencing miRNA (rec-cAAT). pMaxGFP served as a transfection control. Expression from the WT-cAAT cassette was effectively silenced by anti-cAAT miR, while the res-cAAT-expressing construct was not silenced.

Table S1 NHP groups in this study.

	NHP ID	Capsid	Administration Route	Dose	Volume Rate
**Group 1**	1001 Male	AAV3B WT	IV	2.5E13 vg/kg<br>5E13 vg total	1 ml/min
	1501 Female				
	1502 Female				
	1503 Female				
**Group 2**	2001 Male	AAV3B-E13	IV	2.5E13 vg/kg<br>5E13 vg total	1 ml/min
	2002 Male				
	2501 Female				
	2502 Female				
	2503 Female				
**Group 3**	3001 Male	AAV3B-G3	IV	2.5E13 vg/kg<br>5E13 vg total	1 ml/min
	3002 Male				
	3501 Female				
	3502 Female				
	3503 Female				

Table S2. Neutralizing antibodies to AAV3B capsid prior and post dosing. NHP were prescreened for NAbs to the AAV vector before dosing. Only animals with NAb titers below 1:10 were selected. All the animals developed a neutralizing antibody response to AAV3B capsid post dosing (from Day 15 to Day 84).

Vector	Animal ID	Screening	Day 1 (Prior to dose)	Day 15	Day 57	Day 85
AAV3b-WT	1001	1:5	<1:5	1:10240	1:10240	1:2560
	1501	1:5	1:5	1:320	1:160	1:640
	1502	1:5	<1:5	1:80	<1:80	1:80
	1503	1:5	1:10	1:10240	1:160	1:80
AAV3b-E12	2001	1:5	<1:5	>1:20480	>1:20480	1:10240
	2002	1:5	<1:5	1:320	1:1280	1:320
	2501	1:5	1:10	1:320	1:160	1:320
	2502	1:5	<1:5	1:320	1:320	1:160
	2503	1:5	<1:5	1:320	1:160	1:320

**AAV3b-G3**	**3001**	**1:5**	**<1:5**	**1:320**	**1:1280**	**1:1280**
	**3002**	**1:5**	**<1:5**	**1:320**	**1:320**	**1:320**
	**3501**	**<1:5**	**<1:5**	**1:320**	**1:640**	**1:640**
	**3502**	**1:5**	**<1:5**	**1:640**	**1:320**	**1:320**
	**3503**	**1:5**	**<1:5**	**1:160**	**1:320**	**1:160**
