# Supplementary material for: Biodistribution and safety of a single rAAV3B-AAT vector for silencing and replacement of alpha-1 antitrypsin in Cynomolgus macaques
Source: Mol Ther Methods Clin Dev. 2024 Jan 30;32(1):101200. doi: 10.1016/j.omtm.2024.101200 (PMC10914479; doi:10.1016/j.omtm.2024.101200)
Supplement: Document S2. Article plus supplemental information [file mmc2.pdf]

Molecular Therapy
Methods & Clinical Development
Original Article

Biodistribution and safety of a single rAAV3B-AAT vector for silencing and replacement of alpha-1 antitrypsin in *Cynomolgus macaques*

Meghan Blackwood,[1,9] Alisha M. Gruntman,[1,2,3,9] Qiushi Tang,[1] Debora Pires-Ferreira,[1] Darcy Reil,[1] Oleksandr Kondratov,[4] Damien Marsic,[4,5] Sergei Zolotukhin,[4] Gwladys Gernoux,[6] Allison M. Keeler,[1,2,7] Christian Mueller,[8] and Terence R. Flotte[1,2]

[1]Horae Gene Therapy Center, University of Massachusetts Chan Medical School, Worcester, MA 01605, USA; [2]Department of Pediatrics, University of Massachusetts Chan Medical School, Worcester, MA 01605, USA; [3]Department of Clinical Sciences, Cummings School of Veterinary Medicine at Tufts University, North Grafton, MA 01536, USA; [4]Division of Cellular and Molecular Therapy, Department of Pediatrics, University of Florida, Gainesville, FL 32611, USA; [5]MaiBo Biotech, Suzhou Industrial Park, Jiangsu, China; [6]Nantes Université, CHU de Nantes, INSERM, TaRGeT–Translational Research in Gene Therapy, UMR 1089, 44200 Nantes, France; [7]NeuroNexus Institute, University of Massachusetts Chan Medical School, Worcester, MA 01605, USA; [8]Genomic Medicine Unit, Sanofi, Waltham, MA 02451, USA

Alpha-1 antitrypsin deficiency (AATD) is characterized by both chronic lung disease due to loss of wild-type AAT (M-AAT) antiprotease function and liver disease due to toxicity from delayed secretion, polymerization, and aggregation of misfolded mutant AAT (Z-AAT). The ideal gene therapy for AATD should therefore comprise both endogenous Z-AAT suppression and M-AAT overexpression. We designed a dual-function rAAV3B (df-rAAV3B) construct, which was effective at transducing hepatocytes, resulting in a considerable decrease of Z-AAT levels and safe M-AAT augmentation in mice. We optimized df-rAAV3B and created two variants, AAV3B-E12 and AAV3B-G3, to simultaneously enhance the concentration of M-AAT in the bloodstream to therapeutic levels and silence endogenous AAT liver expression in cynomolgus monkeys. Our results demonstrate that AAV3b-WT, AAV3B-E12, and AAV3B-G3 were able to transduce the monkey livers and achieve high M-AAT serum levels efficiently and safely. In this nondeficient model, we did not find downregulation of endogenous AAT. However, the dual-function vector did serve as a potentially "liver-sparing" alternative for high-dose liver-mediated AAT gene replacement in the context of underlying liver disease.

INTRODUCTION

Gene therapy has been considered a promising long-lasting treatment for alpha-1 antitrypsin deficiency (AATD). AAT is the most abundant protease inhibitor in the serum whose main function is to inhibit neutrophil elastase and protect the pulmonary interstitium from excessive proteolytic degradation, particularly during inflammatory responses.[1] AATD is commonly caused by a single mutation (E342K) in the *SERPINA1* gene, which produces a malformed AAT variant (Z-AAT), the Pi*Z phenotype. Hepatocytes are the main source for synthesizing AAT. The misfolded Z-AAT protein aggregates and accumulates within the hepatocytes, resulting in low levels of AAT in the circulation and lungs. This leads to hepatotoxicity and progressive lung disease.[2]

Because AATD is a monogenic disorder, scientists initially believed that a functional AAT (the wild-type version is referred to as AAT-PiM or M-AAT) gene replacement strategy would be sufficient to reverse the disease manifestations. However, clinical studies have failed to show statistically significant improvements, and expression of the transgene has not yet reached the very high therapeutic serum target level (570 μg/mL).[3–5] In addition, M-AAT overexpression does not address AATD liver disease, which is caused by a toxic accumulation of Z-AAT. In fact, functional AAT augmentation gene therapy within hepatocytes without silencing Z-AAT could further advance liver injury.[6,7] Several groups have developed potent small interfering RNA strategies to silence Z-AAT. Among these, Fazisiran has progressed to Phase I clinical trials as a potential therapy for Z-AAT-related liver disease.[8] Individuals with null mutations in *SERPINA1* have typical AAT lung disease, and therefore, silencing of Z-AAT alone without gene replacement or augmentation would not have a therapeutic effect to prevent or treat AAT lung disease, which remains the predominant cause of death in patients with AATD.[2]

Recombinant adeno-associated virus (rAAV) is the main platform for gene delivery for secreted proteins because of their relatively safety, ability to transduce nondividing cells, and capacity to maintain high and sustained expression of the transgene.[9] Thus far, there are seven AAV gene therapy products that have been approved by the US Food and Drug Administration (FDA) and the European

Received 12 September 2023; accepted 24 January 2024;
https://doi.org/10.1016/j.omtm.2024.101200.

[9]These authors contributed equally

Correspondence: Christian Mueller, Genomic Medicine Unit, Sanofi, Waltham, MA 02451, USA.
E-mail: christian.mueller4@sanofi.com
Correspondence: Terence R. Flotte, Horae Gene Therapy Center, University of Massachusetts Chan Medical School, Worcester, MA 01605, USA.
E-mail: terry.flotte@umassmed.edu

This is an open access article under the CC BY license (http://creativecommons.org/licenses/by/4.0/).

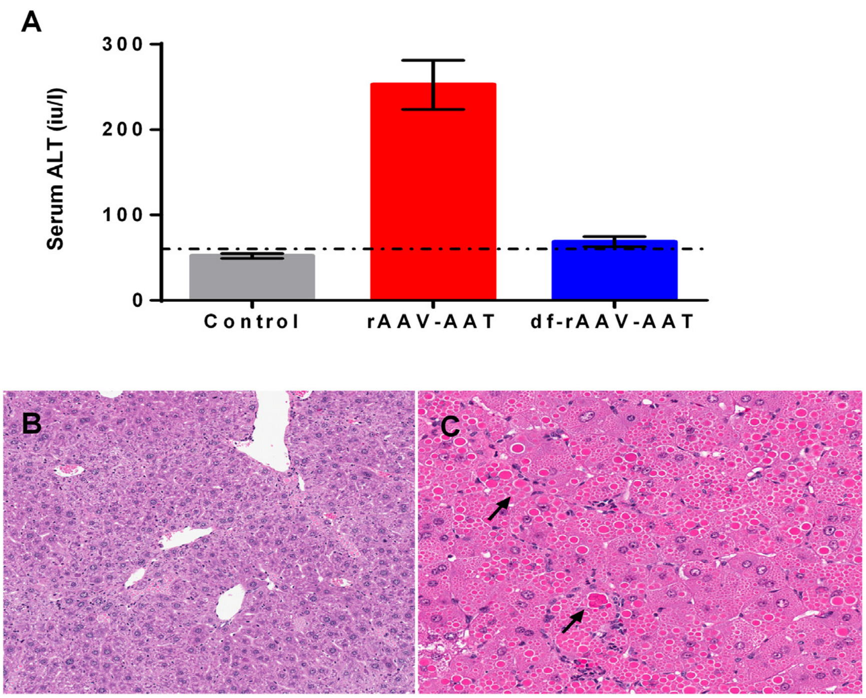

Figure 1. Hepatic toxicity from rAAV-AAT is mitigated by co-expression of synthetic miRNA
(A) Serum ALT levels (a marker of liver injury; mean ± standard error of the mean) are elevated 8 weeks after dosing mice with the standard AAT transgene, but not with the dual-function construct. (B and C) Histopathologic examination of the liver shows more necrosis and large inclusions in the mice treated with the standard transgene (B) than the dual-function vector (C).

Medicines Agency, with an increasing number of new therapies showing promising clinical trial results.[10,11] However, concerns about hepatic toxicity have emerged with various rAAV therapies, most notably in patients with underlying liver disease.[12] As one example, in a recent trial of high-dose (1–3 $\times$ 10^{14} vector genomes [vg]/kg) intravenous (i.v.) rAAV in X-linked myotubular myopathy, a disorder characterized by both muscle and liver involvement, four patients succumbed from liver failure.[12–15] These concerns would clearly apply in using i.v. rAAV to treat AATD lung disease, in which doses greater than 1 $\times$ 10^{14} vg/kg would almost certainly be needed to achieve the very high target therapeutic level. This high level of vector would ideally augment M-AAT while silencing Z-AAT, although this has not been established to be more beneficial than M-AAT augmentation alone.

The rAAV platform has been used in preclinical studies designed to address AATD lung disease while "sparing" the liver from toxicity due to high-level vector expression. Dual-function rAAV (df-rAAV) vectors have been developed to simultaneously silence endogenous Z-AAT and overexpress M-AAT, which is achieved by expressing an M-AAT coding sequence containing silent base mutations, along with a synthetic microRNA (miRNA) designed to silence all endogenous AAT expression (syn-miR). This liver-sparing approach has been shown to lead to significant knockdown of Z-AAT and safe augmentation of M-AAT in PiZ mice following intravenous delivery.[16] Previous studies from our laboratory have demonstrated that rAAV serotype 3B dual-function vector (df-rAAV3B) efficiently transduced human and mouse hepatocytes in a human liver xenograft NSG-PiZ mouse model (1 $\times$ 10^{11} vg/mouse i.v.) and nonhuman primate (NHP) liver (1 $\times$ 10^{13} vg/kg i.v.).[17] AAV3B vectors led to significant liver-targeted expression, which did not reach therapeutic levels as assessed by reporter gene expression, but showed no apparent vector-related hepatotoxicity. Because AAT is the second most abundant secreted serum protein,[2] it is not surprising that achieving a therapeutic level of transgene expression would be a significant challenge. Therefore, approaches to enhance AAV3B transduction efficiency, improve liver tropism, and evade capsid-specific immune responses are required for future clinical application liver-directed AAT gene therapy.[18]

In this study, we sought to first evaluate the liver-sparing property of df-AAV vectors in a human PI*Z-expressing transgenic mouse. We then proceeded to evaluate the safety and efficacy of an optimized version of the rAAV3B dual-function vector (AAV3B-WT [wild type]) as well as two capsid variants, AAV3B-E12 and AAV3B-G3, in an NHP model and examined how well each vector was able to both augment the M-AAT blood levels and knock down endogenous AAT liver production. This approach is similar to that used by Li et al.,[19] but is presented here in the context of an NHP preclinical study intended as a prelude to clinical translation. Our results demonstrate that the i.v. administration of AAV3B-WT, AAV3B-E12, and AAV3B-G3 (2.5 $\times$ 10^{13} vg/kg) was safe. AAV3B-WT and variants were able to achieve a high M-AAT level expression (100 μg/mL) in the serum after 80 days and the liver-targeted AAT augmentation did not cause noticeable hepatotoxicity. However, the vectors were not able to attain significant knockdown of endogenous AAT, and more studies are needed to successfully silence the defective AAT allele while maximizing the expression of a functional allele.

RESULTS

Hepatic toxicity from rAAV vectors expressing WT AAT

Safety concerns due to hepatotoxicity of recombinant AAV vectors in general and particularly in diseases with underlying liver pathology led us to evaluate the potential toxicity of a constitutively high-expressing rAAV vector cassette previously used in muscle-directed human trials in AAT-deficient patients.[3–5,9] This experiment was performed in the well-characterized human PiZ-expressing transgenic mouse model.[24] In PiZ transgenic mice, a serum marker of liver injury, alanine aminotransferase (ALT), was nearly 5-fold higher at 12 weeks postinjection in mice treated with the standard rAAV8-AAT vector (Figure 1A) as compared with the previously described df-rAAV8 vector.[16] Histopathologic

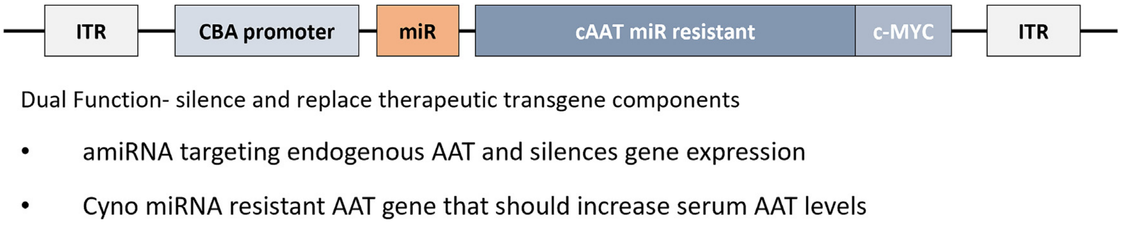

Figure 2. Design of the dual-function vector containing a CBA promoter, a syn-miR sequence complementary to regions of the AAT gene-coding sequence, an AAT gene containing silent mutations to induce mismatches with the miRNA, and a c-MYC tag

examination confirmed a greater extent of hepatocellular necrosis and large inclusions in the animals receiving the standard rAAV8-AAT vector compared to the dual-function vector (Figures 1B and 1C).

Design of df-rAAV constructs for safety studies in NHPs

We designed a df-rAAV3B vector consisting of a construct carrying both a miRNA guide that can base pair with endogenous AAT mRNA to inhibit its translation and expression and a miRNA-resistant version of the cynomolgus M-AAT gene, which contained silent mutations intended to induce mismatches in base pairing with the expressed miRNA (Figure 2). The cynomolgus M-AAT was also tagged with a c-MYC epitope tag to allow for distinguishing vector-mediated M-AAT expression from endogenous cynomolgus AAT gene expression.

Design of NHP study to define biodistribution and safety with each of three rAAV3B capsid variants

In anticipation of the use of the df-rAAV vector in clinical trials, we designed a biodistribution and safety study to be performed in 14 cynomolgus macaques (Table S1). Capsid serotypes chosen for this trial were derived from AAV3B, which has previously been shown to be particularly effective in NHP liver.[17] Five NHPs were assigned to receive the dual-function vector packaged in the AAV3B-E12 or AAV3B-3G capsid (three female, two male in each group), whereas four received it packaged in the parental AAV3B capsid (three female, one male). Each animal received 2.5×10^{13} vg/kg of the vector. Samples from untreated animals were used for reference controls. Screening for preexisting neutralizing antibodies (NAbs) as well as antibodies generated during the course of the study indicated that the animals were effectively seronegative before delivery, and each demonstrated a strong humoral immune response to the capsids (Table S2).

Biodistribution and safety of df-rAAV3B vectors

The animals were sacrificed at 90 days after i.v. infusion of df-rAAV, and the biodistribution of rAAV genomes to peripheral organs was assessed by droplet digital (dd)PCR. As expected, the greatest abundance of rAAV genomes was observed in the liver, at levels of $\sim$100 vg per diploid genome (Figure 3). The biodistribution to liver and other organs was similar across the groups, except for greater biodistribution to nonhepatic organs (including heart, lungs, spinal cord, and gonads) by the rAAV3B-G3 group (Figures 3 and S1). The overall safety assessment was positive, with no adverse events noted in study animals. Laboratory assessments of liver enzymes (ALT, aspartate aminotransferase [AST]) and blood count values (platelet count, white blood cell count, and blood hemoglobin concentration) showed no differences among the groups nor any consistent deviations from the normal range (Figure 4). Histological analysis of the liver revealed no abnormalities and confirmed the delivery and expression of the transgene to hepatocytes by c-MYC immunofluorescence (Figure 5). Minimal background fluorescence was observed when liver tissue from an uninjected animal was stained (Figure S2).

Quantification of transgene expression

The levels of vector-mediated M-AAT expression were determined by quantitative western blot analysis on serum samples obtained at baseline and 21, 45, and 90 days after vector infusion. As demonstrated in Figure 6, the expression level from NHPs in each of the groups approached 100 μg/mL. These levels were reached on day 21 and maintained through days 45 and 90. Although there were no statistically significant differences among the groups, the parental AAV3B and AAV3b-G3 capsid groups showed a trend toward higher expression.

Biological activity of syn-miRNA expression

No significant differences were noted in the expression of the endogenous cynomolgus M-AAT gene among the three experimental groups, and the endogenous mRNA levels were similar to those in parallel control NHP samples. A second allele-specific PCR primer set was designed (Figure S3), which confirmed this finding (Figure 7). The expression of the syn-miRNA was then assayed by qPCR. The syn-miRNA was found to be expressed in the livers of all of the animals in each of the three groups, with no significant differences among them (Figure 8). In our preliminary *in vitro* studies, we observed appreciable knockdown of endogenous AAT protein levels secreted into the media as well as in cell lysates (Figure S4).

DISCUSSION

In this report, we evaluated the safety, bioactivity, and biodistribution of rAAV3B-dfAAT vectors in NHPs as a step in their clinical development. We compared the parental AAV3B capsid to two variants and found that all of them were able to produce clinically relevant levels of AAT augmentation in the serum. The three vectors showed a favorable safety profile while sustaining a high level of expression of synthetic miRNAs in the primate liver. Although we had initially hoped that the newer AAV3B variants would perform better with respect to vector expression, the lack of correlation between different species has been described previously, and likely relates to relative

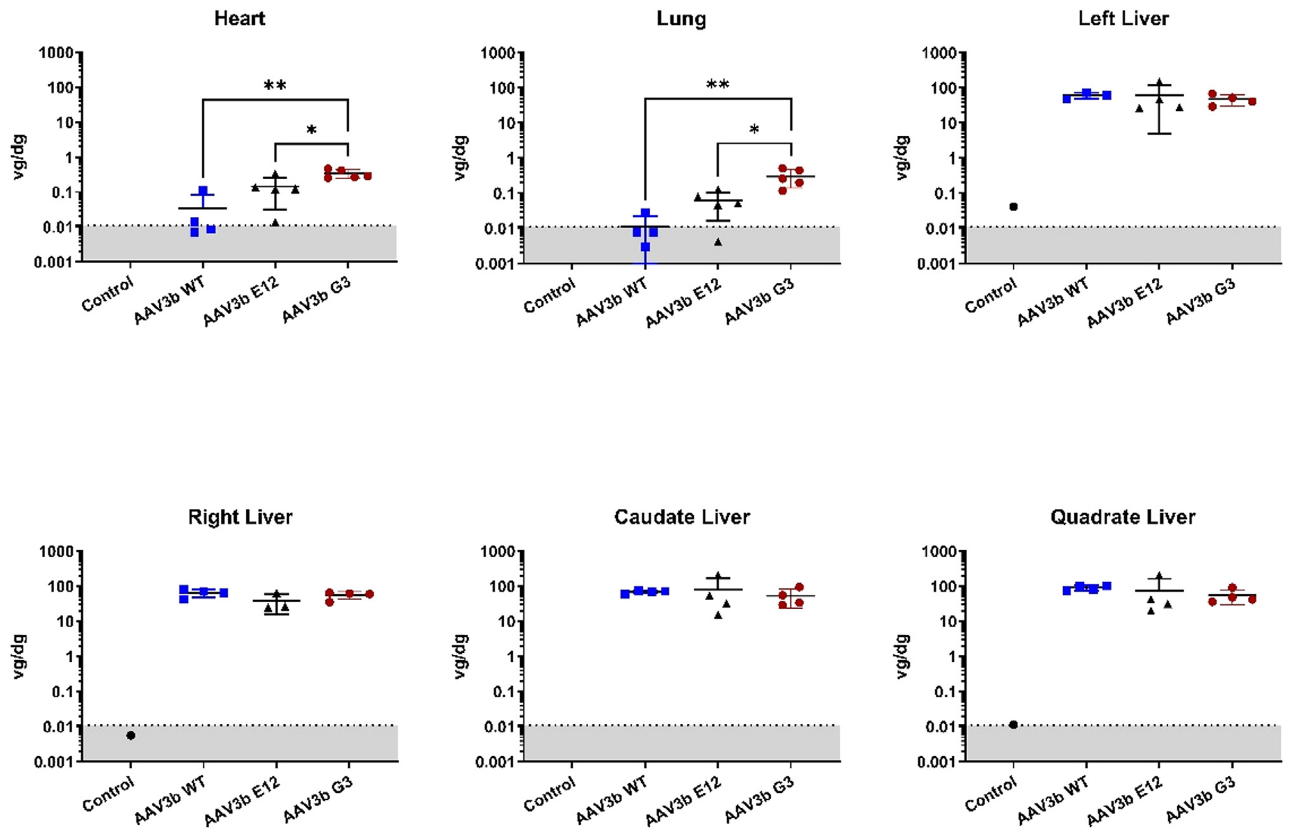

Figure 3. The AAV3B-G3 capsid showed higher concentration in the heart and lung than the WT and E12 capsids, but no difference in the liver
The lines and error bars indicate mean ± standard deviation; *$p < 0.05$, **$p < 0.01$ determined by 1-way ANOVA, followed by Tukey post hoc test.

receptor abundance.[26,27] The lack of hepatotoxicity in NHP liver with constitutive expression of an AAT-directed syn-miR stands in contrast to several reports of cytotoxicity of rAAV-syn-miR vectors in the brain and spinal cord of NHPs.[26,29] In addition, the safety of constitutive, unregulated expression of the df-AAT differs from expression-silencing approaches that require precise regulation, as with the miR-regulated AAV9miniMECP2 vector in Rett syndrome mice.[25] The present study, along with other recent proof-of-concept studies of the silence-and-replace strategy with rAAV,[23] would support further attempts at the clinical development of df-rAAV therapies.

AATD caused by the most common E342K mutation presents with both lung disease due to a lack of AAT function and liver disease due to hepatocyte accumulation of toxic misfolded AAT (Z-AAT) aggregates. Therefore, an ideal therapy for AATD should simultaneously cause knockdown of endogenous Z-AAT and overexpression of vector-delivered M-AAT.[7] This approach appears to be feasible from our initial observation in this study that the df-rAAV vector caused less liver toxicity in the Z-AAT transgenic mouse model than the monofunctional rAAV-AAT expression vector. Next, we chose the cynomolgus monkey as a useful large-animal model for toxicological and pharmacological evaluations because of their similarity to humans with respect to vector tropism and physiology.[28] Efforts have been made recently to enhance AAV transduction efficiency.

We also packaged the vector into two rAAV3B capsid variants (AAV3B-E12 and AAV3B-G3), which were identified in screens for capsid variants with enhanced hepatotropism[18,30] to assess whether they are able to enhance the efficiency of df-rAAV in these NHPs. To further enhance M-AAT expression, we added a *cis*-regulatory module enhancer element to the chicken β-actin (CBA) promoter region in our previously published rAAV3B construct, increasing gene expression and secretion more than 100-fold, as determined by an AAT ELISA in tissue culture supernatants of transfected cells.[17] In addition, we eliminated CpG motifs that could be proinflammatory.[31,33] Based on earlier findings in a murine model, we replaced the three miRNAs against AAT with a single miRNA in an effort to achieve significant knockdown of endogenous AAT. In addition, we introduced GC base substitutions to reduce anti-AAV capsid immune responses that may limit transgene expression. A significant positive finding in this study was that AAV3b-WT, AAV3B-E12, and AAV3B-G3 (2.5×10^{13} vg/kg) were each able to achieve a high level (∼100 μg/mL) of myc-AAT in the serum (Figure 6), which is nearly 20% of the level associated with lung protection (570 μg/mL).[2,32] This suggests that a 6-fold increase in dose (1.5×10^{14} vg/kg) with this construct should reach the human therapeutic target of serum

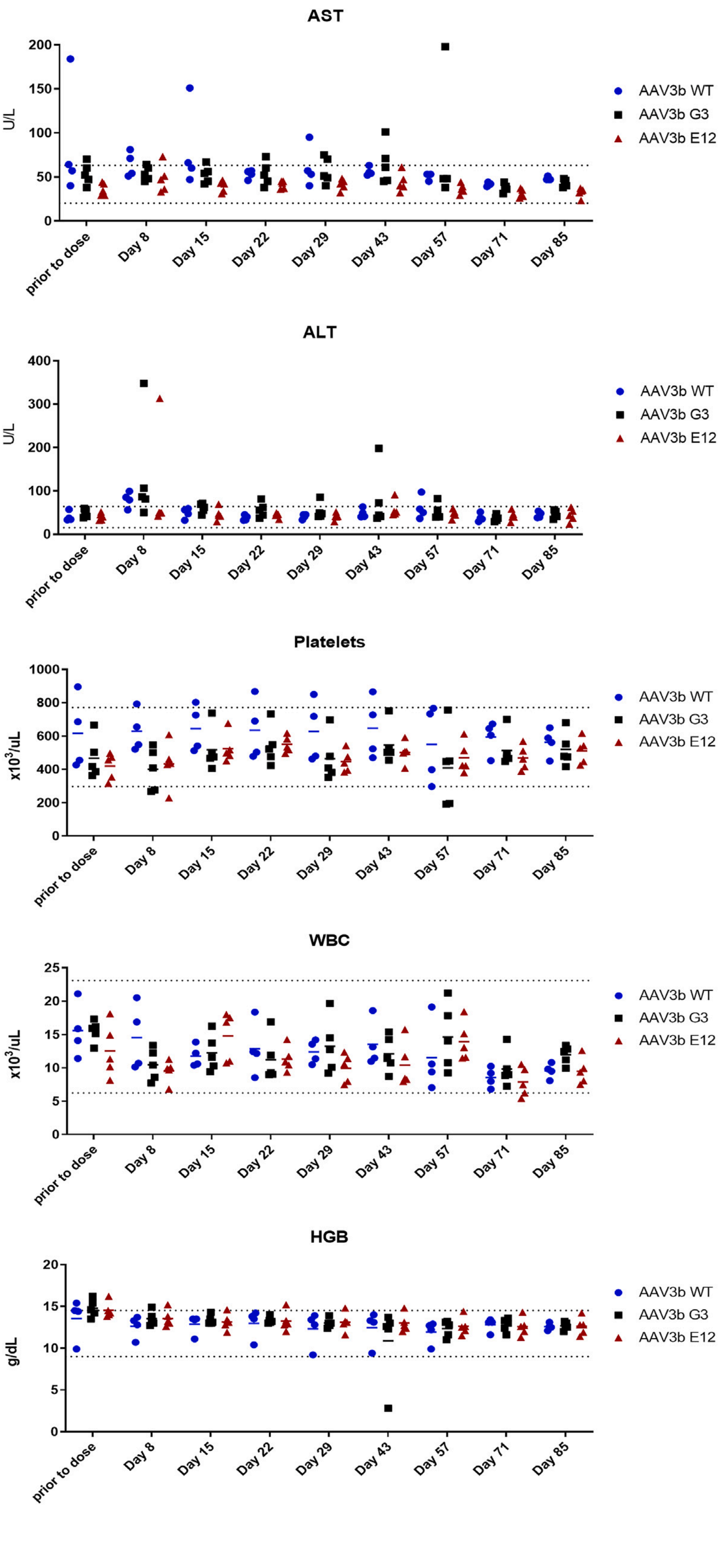

Figure 4. Circulating AST and ALT levels, as well as platelet count, white blood cell (WBC) count, and blood hemoglobin (HGB) concentration were within normal ranges and showed no significant differences among the groups during the study. Lines indicate means.

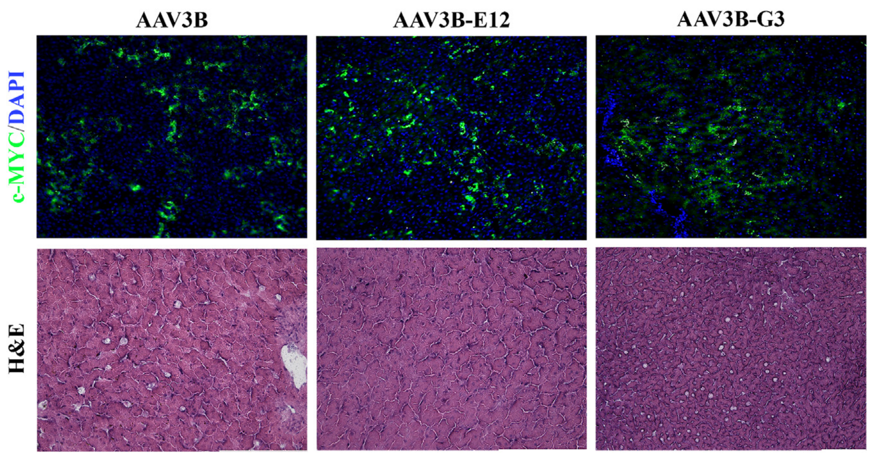

Figure 5. c-MYC staining indicates that the transgene was successfully expressed in the liver in all 3 groups, and no abnormalities were found in the liver after histopathological examination
Green: c-MYC; blue: DAPI.

AAT concentration. This is a feasible dose level that has been used in rAAV-based gene therapies for Duchenne muscular dystrophy (DMD) and spinal muscular atrophy, which are now both FDA approved.[34,35]

A significant limitation of this study is that the rAAV3B vectors used in this NHP study did not mediate a significant reduction in the expression of the endogenous cynomolgus AAT gene at the doses given. The reason for this finding is not clear. Our construct is designed to deliver the syn-miR by expressing it as a portion of the RNA polymerase II (RNA Pol II) promoter transcript that also drives the AAT cDNA expression, as originally described by Stegmeier and as used in our murine model work.[16,36] In prior studies, processing of such syn-miRs from the RNA Pol II transcript was efficient enough to effectively silence the Z-AAT allele in the transgenic mouse model, a model that has integrated copies of the entire human *SERPINA1* locus, including the endogenous human AAT promoter. The fact that levels of the syn-miR were statistically significant indicates that the primary issue was not a complete failure to process the syn-miR from the vector transcript. It may demonstrate that the level of expression from the endogenous AAT promoter was so great that the syn-miR could not appreciably decrease the steady-state AAT mRNA levels. One way that this could occur is if the vector administration or the miR stimulated expression from the endogenous AAT promoter, which is known to be induced by various signals, including interleukin-6 and oncostatin.[37] In fact, the responsivity of the AAT promoter as an acute phase reactant could have affected several of the outcomes in this study, including the slight increase in endogenous AAT levels seen in Figure 7. Therefore, one additional approach to be considered for vector development would be to express the syn-miR from a highly active RNA Pol III promoter such as U6 or H1, as we have done in other contexts.[38]

Other future improvements in a clinical stage df-rAAV vector may entail optimizing the choice of AAV capsid and promoter elements. Studies have shown that of the many AAV serotypes isolated, AAV8 has the greatest affinity for liver transduction and expression in mice.[39] Nathwani et al. demonstrated that the AAV8 vector resulted in clinically effective liver transfer and significant levels of factor IX in hemophilia B patients.[40] Despite the potential advantage of AAV8 over AAV5,[41] the high prevalence of preexisting Nabs against the AAV8 capsid in humans may limit its application for liver-directed gene therapy.[42] To overcome the capsid-specific immune responses and enhance hepatocyte tropism, scientists have tried to find new serotypes and engineer current AAVs to achieve efficient, sustained, and safe liver transduction.[43,44] In a previous study from our laboratory with a humanized liver xenograft mouse model, AAV3B was ~12-fold more efficient than AAV8 in transducing human hepatocytes *in vivo* because it uses the human hepatocyte growth factor receptor as a cellular co-receptor for viral entry, leading to successful liver-targeted gene transfer.[27] The i.v. injection of the AAV3B viral vectors led to liver-directed delivery, with relatively low viral distribution to other organs. This may ultimately prove to be useful in a variety of gene therapies.

Taken together, our results indicate that AAV3b-WT, AAV3B-E12, and AAV3B-G3 viral vectors are safe and efficient in delivering the M-AAT gene to the liver of this NHP model, as well as driving high serum levels of the AAT protein. Further optimization may be required to achieve a level of silencing required to treat AAT liver disease, but the liver-sparing design of df-AAV vectors remains a very promising option for safely targeting the liver for AAT gene replacement. Other alternatives for the future development of a liver-sparing gene therapy for AAT could rely on gene editing, base editing, and prime editing approaches. CRISPR-Cas9-mediated homology-directed repair of the E342K mutation in the *SERPINA1* gene has recently been accomplished in mouse models.[45] More recently, the use of a dCas9-based adenine base editor has shown very promising results in correction of the E342K mutation, as has a prime editing approach.[45,46] As with dual-function approaches, the bioefficacy and safety of each of these approaches will ultimately have to be confirmed in large-animal models as a prelude to their use in clinical trials.

MATERIALS AND METHODS

AAV vector constructs, production, and purification

For these studies, the AAV capsids used included AAV3B and two variants with amino acid substitutions generated from *in vivo* selection in humanized NSG-PiZ mice previously used in AAV3B characterization.[17] AAV3B-G3 contains 15 amino acid substitutions as compared with parental AAV3B (G449S, T451A, N457G, Q458T, R460T, L462R, S386N, S587G, N588R, T589D, A590N, T593F, T595D, N597Q, and D598H). AAV3B-E12 has 24 mutations including 1 amino acid deletion (Δ D178) and 23 substitutions (S268T, G449S, T451A, N457G, Q458T, R460T, L462R, T492I,

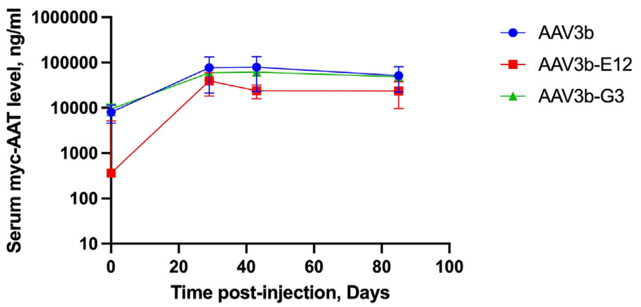

Figure 6. Concentration of c-MYC-tagged AAT in the serum increased in all groups and was maintained through the course of the study at nearly 100 μg/mL

These levels are ~20% of the therapeutic threshold (570,000 ng/mL). Data are mean ± standard error of the mean.

A493P, N494G, D495Q, K508T, E531D, K533R, E546Q, G547D, T549A, A550R, N552D, A553D, L555V, D556G, and N557K). Vectors were packaged into each of the 3 capsids, purified, and characterized as previously described.[20,21]

Pilot murine safety study

All of the experimental procedures were approved by the Institutional Animal Care and Use Committee at the University of Massachusetts Chan Medical School. PiZ (*Serpina1*$^{E342K/E342K}$) mice were housed in groups of 5 on a 12-h light cycle and had free access to water, were fed a standard mouse chow *ad libitum*, and were bred in-house. Five 5-week-old male mice were dosed with 1.0×10^{12} vector particles by tail-vein injection of either rAAV8-CB-AAT (a vector constitutively expressing human M-AAT) or rAAV8-dfAAT (a vector construct expressing a dual-function cassette expressing both an anti-hAAT syn-miR and an M-AAT coding sequence, with silent base changes rendering it resistant to the syn-miR).[16] At 8 weeks post-injection, mice were sacrificed, serum was collected for measurement of AST, and the livers were collected for histopathology. Livers were fixed in 4% (v/v) formalin overnight and embedded in paraffin. Tissues were sectioned (4 μm) and stained with H&E.

NHP animal protocol

NHP studies were conducted at the Northern Biomedical Research testing facility following approval by the Institutional Animal Care and Use Committees of Northern Biomedical Research and the University of Massachusetts Chan Medical School (study no. 088-005). NHPs were being prescreened for Nabs to the AAV vector before dosing, and only animals with Nab titers below 1:10 were selected. Cynomolgus monkeys were dosed with 2.5×10^{13} vg/kg of df-AAV3B (1 male, 3 female), df-AAV3B-E12 (2 male, 3 female), or df-AAV3B-G3 (2 male, 3 female) by i.v. administration (Table S1). Depo-medrol was administered intramuscularly at days −2 (40 mg), 7 (40 mg), and 14 (20 mg). Blood samples were collected and body weights were recorded before dosing and then weekly for 12 weeks, with complete blood counts and chemistries performed by the clinical veterinary laboratory on site. At 85 days postdosing, animals were sacrificed and tissue samples were collected (brain, spinal cord, kidneys, liver, spleen, heart, pancreas, lungs, quadriceps muscle, ovaries/testes, and lymph nodes). Blood samples were collected and divided. Part of the sample was collected in tubes containing heparin for whole-blood analysis. The remaining part was collected in tubes without anticoagulants, stored at room temperature (RT) until clotted, and then centrifuged at 1,500 × *g* to obtain serum for quantitative western blot analysis of gene expression.

c-MYC immunofluorescence staining

Frozen liver tissue was sectioned on a cryostat microtome (10 μm thick) and mounted onto glass slides. Sections were fixed in 4% paraformaldehyde for 15 min at RT. The slides were then washed 4 times in PBS for 5 min each and then blocked/permeabilized in 20% donkey serum + 0.001% Tween 20 in PBS for 3 h at RT. The samples were subsequently incubated with goat c-MYC primary antibody (GeneTex catalog no. GTX30518), diluted in 5% donkey serum (1:250), at 4°C in the dark overnight. The next day, the slides were washed 4 times in PBS for 5 min each and then incubated with the secondary antibody (donkey anti-goat immunoglobulin G [IgG] H&L [Alexa Fluor 488], Abcam catalog no. 150129), diluted in 5% donkey serum (1:500), and incubated for 1 h in the dark at RT. The slides were washed in PBS 5 times for 5 min each, and then the nuclei were stained by incubation with 'DAPI solution (1:10,000) for 5 min at RT in the dark. Finally, the slides were washed 2 times in PBS for 5 min each, left to dry overnight, and then mounted. The slides were examined using a fluorescence microscope at 40× magnification (Leica Thunder).

Quantification of transgene expression by western blot

Western blots were done as previously described.[22] Serum samples and standards were diluted 1:50 in PBS. Then, 10 μL diluted serum was mixed with 10 μL 2× Novex Tris-glycine SDS sample buffer (Invitrogen, Carlsbad, CA) and heated at 85°C for 10 min. Samples were run on Novex 12% Tris-glycine gels (Invitrogen) using Tris-glycine SDS running buffer (Invitrogen). Proteins were then transferred to nitrocellulose membranes using an i-Blot transfer device (Invitrogen). Membranes were blocked for 1 h at RT with Odyssey Blocking Buffer (LI-COR Biosciences, Lincoln, NE) before being probed overnight with primary antibodies (1:1,000 dilution goat c-MYC antibody; GeneTex catalog no. 30518). Infrared (IR) dye-labeled secondary antibodies (1:5,000 dilution) were then applied using IRDye 680LT donkey anti-goat IgG (H&L). Blots were visualized using the Odyssey Infrared imaging system (LI-COR Biosciences), and images were processed using an image studio program. All of the antibodies were used at the manufacturer-recommended dilutions.

Measurement of vector-mediated synthetic miRNA expression

Quantitative reverse transcriptase-PCR analysis was done as previously described.[23] A TaqMan miRCURY LNA SYBR Green PCR kit (Qiagen 33945, Qiagen, Hilden, Germany) was used to measure the expression of miRNA (customer-designed miRCURY LNA miRNA assay, Qiagen). U6 snRNA was used as an internal control (Qiagen assay YP02119464). Total RNA was extracted using QIAzol (Qiagen), followed by cDNA synthesis using a miRCURY LNA RT (reverse transcriptase) Kit (Qiagen 339340). qPCR was performed

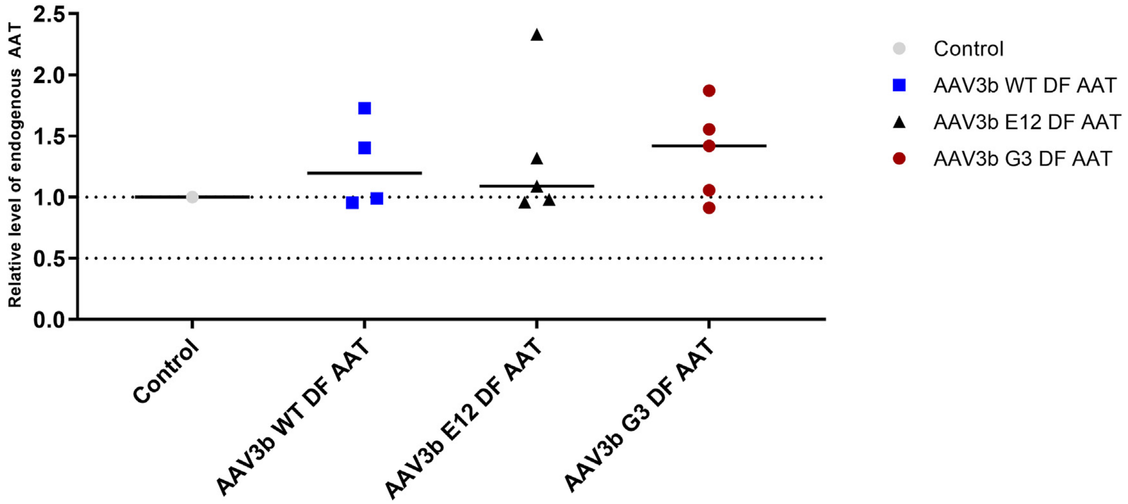

Figure 7. Endogenous AAT levels were not reduced in the serum after dosing with the dual-function vector in any of the groups, indicating that the expressed miRNA was not able to silence expression of the gene. Lines indicate median values.

using a CFX Connect reverse transcriptase-PCR detection system (Bio-Rad).

Biodistribution of vector genomes

Genomic DNA was extracted from frozen tissues using the Gentra Puregene Tissue kit (Qiagen). Vector biodistribution of peripheral organs was measured using ddPCR according to the manufacturer's recommendations using 50 ng genomic DNA as input.

Evaluation of endogenous AAT expression

RNA extracted using TRIzol (Invitrogen) was retrotranscribed using a high-capacity RNA-to-cDNA kit (Applied Biosystems, Waltham, MA). A custom assay (fluorescein amidite -labeled primer/probe) was designed to target the endogenous cynomolgus SERPINA1 (Bio-Rad), and ddPCR reactions were prepared and run according to the manufacturer's instructions. The data are expressed relative to cynomolgus albumin and normalized by setting the average of the control group at 1. Each run had to have at least 10,000 accepted droplets, of which 100 were negative to be considered valid.

Cell culture and transfection

HEK293 cells were cultured in DMEM supplemented with 10% fetal bovine serum and 100 mg/L of penicillin-streptomycin (Gemini Bio-products, West Sacramento, CA). Cells were maintained in a humidified incubator at 37°C and 5% CO_2. Plasmids were transiently transfected using Lipofectamine LTX and plus reagent (Invitrogen) according to the manufacturer's instructions.

HEK293 cells were cotransfected with a plasmid containing AAT cDNA (cAAT) or a version that is resistant to miRNA silencing (res-cAAT), as well as a plasmid containing the silencing miRNA (anti-cAAT-miR-GFP) or a PBS control. The transfected cells were incubated for 72 h, the cells and the culture supernatant were collected, and the cells were lysed.

Human AAT ELISA

High binding extra 96-well plates (Immulon 4; Dynatech Laboratories, Chantilly, VA) were coated with 100 μL of human-specific goat anti-AAT (1:2,000 diluted; Bethyl Laboratories, Montgomery, TX) in Voller's buffer overnight at 4°C. After blocking with 1% nonfat dry milk in PBS with Tween 20 (PBST), duplicate standard curves (Athens Research and Technology, Athens, GA) and serially diluted experimental samples were incubated in the plate at RT for 1 h, and a second goat anti-hAAT (horseradish peroxidase) antibody (1:5,000 dilution; Bethyl Laboratories) was incubated at RT for 1 h. The plate was washed with PBST between reactions. After reaction with 3,3′, 5,5′ *tetramethylbenzidine* dihydrochloride peroxidase substrate (KPL, Gaithersburg, MD), reactions were stopped by adding 2 N H_2SO_4 (Fisher Scientific, Hudson, NH). Plates were read at 450 nm on a VersaMax microplate reader (Molecular Devices, Sunnyvale, CA). The cell culture, transfection, and ELISA studies were performed as previously described.[16]

DATA AND CODE AVAILABILITY

All of the data are available from the corresponding author upon reasonable request.

SUPPLEMENTAL INFORMATION

Supplemental information can be found online at https://doi.org/10.1016/j.omtm.2024.101200.

ACKNOWLEDGMENTS

The authors acknowledge Dr. Thomas Gallagher for his able assistance in the preparation of this manuscript. The authors are thankful to Dr. Christen Simon and the staff at Northern Biomedical Research for assistance in carrying out the NHP studies. This research was supported by the National Heart, Lung, and Blood Institute (NHLBI) of the NIH (award nos. P01HL158506 and P01HL131471). T.R.F. and

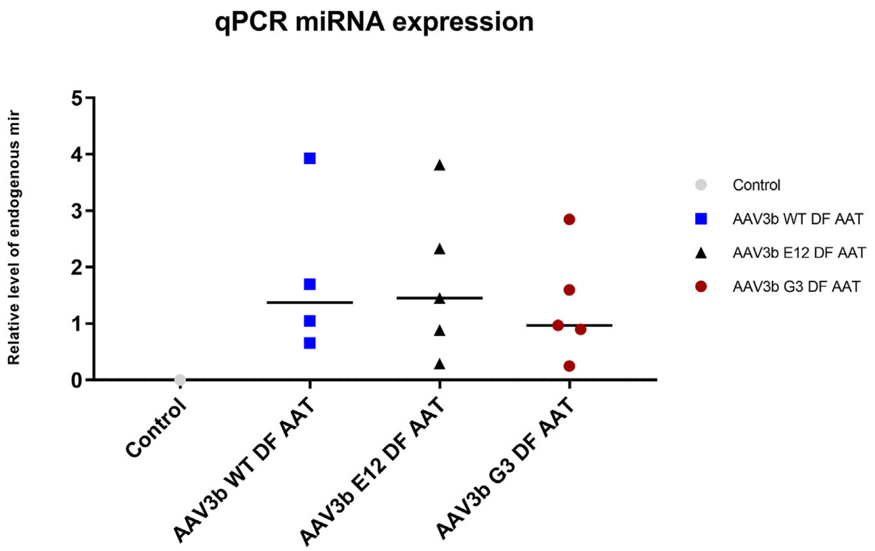

Figure 8. Expression of the miRNA in the liver was validated by qPCR, and was found to be expressed in all 3 groups. Lines indicate median values.

A.G. report equipment, drugs, or supplies were provided by the NHLBI Gene Therapy Resource Program.

AUTHOR CONTRIBUTIONS

C.M. and T.R.F. conceived of the project and acquired the funding. M.B., A.M.G., Q.T., D.P.F., D.R., and G.G. curated the data. M.B., A.M.G., Q.T., D.P.F., D.R., and G.G. performed the investigation. M.B., A.M.G., Q.T., D.P.F., D.R., G.G., A.K., and T.R.F. analyzed the data. A.M.G., O.K., D.M., S.Z., and T.R.F. provided resources for this project. A.M.G., G.G., A.K., C.M., and T.R.F. supervised this project. M.B., A.M.G., Q.T., D.P.F., D.R., and T.R.F. visualized the data. A.M.G. and T.R.F. wrote the manuscript. M.B., A.M.G., Q.T., D.P.F., D.R., and T.R.F. reviewed and edited the manuscript.

DECLARATION OF INTERESTS

T.R.F., A.G., C.M., and A.K. report financial support was provided by National Heart Lung and Blood Institute. T.R.F. and A.G. report equipment, drugs, or supplies was provided by National Heart Lung and Blood Institute Gene Therapy Resource Program. C.M. reports a relationship with Sanofi that includes: employment. C.M. reports a relationship with Apic Bio that includes: board membership and equity or stocks. T.R.F. and C.M. have a patent pending to Apic Bio. T.R.F. is Chair of the NHLBI Gene Therapy Resource Program Advisory Committee. C.M. is a named inventor on the patent for the dual function AAT construct; D.M., S.Z., and C.M. are named inventors on the patent for the AAV3B variants.

REFERENCES

1. Sapey, E. (2020). Neutrophil Modulation in Alpha-1 Antitrypsin Deficiency. J COPD F *7*, 247–259. https://doi.org/10.15326/jcopdf.7.3.2019.0164.
2. Strnad, P., McElvaney, N.G., and Lomas, D.A. (2020). Alpha1-Antitrypsin Deficiency. N. Engl. J. Med. *382*, 1443–1455. https://doi.org/10.1056/NEJMra1910234.
3. Brantly, M.L., Chulay, J.D., Wang, L., Mueller, C., Humphries, M., Spencer, L.T., Rouhani, F., Conlon, T.J., Calcedo, R., Betts, M.R., et al. (2009). Sustained transgene expression despite T lymphocyte responses in a clinical trial of rAAV1-AAT gene therapy. Proc. Natl. Acad. Sci. USA *106*, 16363–16368. https://doi.org/10.1073/pnas.0904514106.
4. Flotte, T.R., Trapnell, B.C., Humphries, M., Carey, B., Calcedo, R., Rouhani, F., Campbell-Thompson, M., Yachnis, A.T., Sandhaus, R.A., McElvaney, N.G., et al. (2011). Phase 2 Clinical Trial of a Recombinant Adeno-Associated Viral Vector Expressing α_1 -Antitrypsin: Interim Results. Hum. Gene Ther. *22*, 1239–1247. https://doi.org/10.1089/hum.2011.053.
5. Mueller, C., Chulay, J.D., Trapnell, B.C., Humphries, M., Carey, B., Sandhaus, R.A., McElvaney, N.G., Messina, L., Tang, Q., Rouhani, F.N., et al. (2013). Human Treg responses allow sustained recombinant adeno-associated virus–mediated transgene expression. J. Clin. Invest. *123*, 5310–5318. https://doi.org/10.1172/JCI70314.
6. Rahaghi, F.F. (2021). Alpha-1 antitrypsin deficiency research and emerging treatment strategies: what's down the road? Therapeutic Advances in Chronic Disease *12*, 204062232110140. https://doi.org/10.1177/20406223211014025.
7. Pires Ferreira, D., Gruntman, A.M., and Flotte, T.R. (2023). Gene therapy for alpha-1 antitrypsin deficiency: an update. Expet Opin. Biol. Ther. *23*, 283–291. https://doi.org/10.1080/14712598.2023.2183771.
8. Strnad, P., Mandorfer, M., Choudhury, G., Griffiths, W., Trautwein, C., Loomba, R., Schluep, T., Chang, T., Yi, M., Given, B.D., et al. (2022). Fazirsiran for Liver Disease Associated with Alpha $_1$ -Antitrypsin Deficiency. N. Engl. J. Med. *387*, 514–524. https://doi.org/10.1056/NEJMoa2205416.
9. Mueller, C., Gernoux, G., Gruntman, A.M., Borel, F., Reeves, E.P., Calcedo, R., Rouhani, F.N., Yachnis, A., Humphries, M., Campbell-Thompson, M., et al. (2017). 5 Year Expression and Neutrophil Defect Repair after Gene Therapy in Alpha-1 Antitrypsin Deficiency. Mol. Ther. *25*, 1387–1394. https://doi.org/10.1016/j.ymthe.2017.03.029.
10. Keeler, A.M., and Flotte, T.R. (2019). Recombinant Adeno-Associated Virus Gene Therapy in Light of Luxturna (and Zolgensma and Glybera): Where Are We, and How Did We Get Here? Annu. Rev. Virol. *6*, 601–621. https://doi.org/10.1146/annurev-virology-092818-015530.
11. Au, H.K.E., Isalan, M., and Mielcarek, M. (2021). Gene Therapy Advances: A Meta-Analysis of AAV Usage in Clinical Settings. Front. Med. *8*, 809118. https://doi.org/10.3389/fmed.2021.809118.
12. Wilson, J.M., and Flotte, T.R. (2020). Moving Forward After Two Deaths in a Gene Therapy Trial of Myotubular Myopathy. Hum. Gene Ther. *31*, 695–696. https://doi.org/10.1089/hum.2020.182.
13. Mendell, J.R., Al-Zaidy, S.A., Rodino-Klapac, L.R., Goodspeed, K., Gray, S.J., Kay, C.N., Boye, S.L., Boye, S.E., George, L.A., Salabarria, S., et al. (2021). Current Clinical Applications of In Vivo Gene Therapy with AAVs. Mol. Ther. *29*, 464–488. https://doi.org/10.1016/j.ymthe.2020.12.007.

14. Shieh, P.B., Bönnemann, C.G., Müller-Felber, W., Blaschek, A., Dowling, J.J., Kuntz, N.L., and Seferian, A.M. (2020). Re: "Moving Forward After Two Deaths in a Gene Therapy Trial of Myotubular Myopathy" by Wilson and Flotte. Hum. Gene Ther. *31*, 787. https://doi.org/10.1089/hum.2020.217.

15. Shieh, P., Kuntz, N., Dowling, J., Müller-Felber, W., Bönnemann, C., Foley, D., Seferian, A., Servais, L., Lawlor, M., Noursalehi, M., et al. (2022). OP018: ASPIRO gene therapy trial in X-Linked Myotubular Myopathy (XLMTM): Update on preliminary efficacy and safety findings. Genet. Med. *24*, S350. https://doi.org/10.1016/j.gim.2022.01.568.

16. Mueller, C., Tang, Q., Gruntman, A., Blomenkamp, K., Teckman, J., Song, L., Zamore, P.D., and Flotte, T.R. (2012). Sustained miRNA-mediated Knockdown of Mutant AAT With Simultaneous Augmentation of Wild-type AAT Has Minimal Effect on Global Liver miRNA Profiles. Mol. Ther. *20*, 590–600. https://doi.org/10.1038/mt.2011.292.

17. Li, S., Ling, C., Zhong, L., Li, M., Su, Q., He, R., Tang, Q., Greiner, D.L., Shultz, L.D., Brehm, M.A., et al. (2015). Efficient and Targeted Transduction of Nonhuman Primate Liver With Systemically Delivered Optimized AAV3B Vectors. Mol. Ther. *23*, 1867–1876. https://doi.org/10.1038/mt.2015.174.

18. Biswas, M., Marsic, D., Li, N., Zou, C., Gonzalez-Aseguinolaza, G., Zolotukhin, I., Kumar, S.R.P., Rana, J., Butterfield, J.S.S., Kondratov, O., et al. (2020). Engineering and In Vitro Selection of a Novel AAV3B Variant with High Hepatocyte Tropism and Reduced Seroreactivity. Mol. Ther. Methods Clin. Dev. *19*, 347–361. https://doi.org/10.1016/j.omtm.2020.09.019.

19. Li, C., Xiao, P., Gray, S.J., Weinberg, M.S., and Samulski, R.J. (2011). Combination therapy utilizing shRNA knockdown and an optimized resistant transgene for rescue of diseases caused by misfolded proteins. Proc. Natl. Acad. Sci. USA *108*, 14258–14263. https://doi.org/10.1073/pnas.1109522108.

20. Ayuso, E., Mingozzi, F., Montane, J., Leon, X., Anguela, X.M., Haurigot, V., Edmonson, S.A., Africa, L., Zhou, S., High, K.A., et al. (2010). High AAV vector purity results in serotype- and tissue-independent enhancement of transduction efficiency. Gene Ther. *17*, 503–510. https://doi.org/10.1038/gt.2009.157.

21. Sena-Esteves, M., and Gao, G. (2020). Introducing Genes into Mammalian Cells: Viral Vectors. Cold Spring Harb. Protoc. *2020*, 095513. https://doi.org/10.1101/pdb.top095513.

22. Gruntman, A.M., Gernoux, G., Tang, Q., Ye, G.-J., Knop, D.R., Wang, G., Benson, J., Coleman, K.E., Keeler, A.M., Mueller, C., et al. (2019). Bridging from Intramuscular to Limb Perfusion Delivery of rAAV: Optimization in a Non-human Primate Study. Mol. Ther. Methods Clin. Dev. *13*, 233–242. https://doi.org/10.1016/j.omtm.2019.01.013.

23. Yang, Y.-S., Kim, J.-M., Xie, J., Chaugule, S., Lin, C., Ma, H., Hsiao, E., Hong, J., Chun, H., Shore, E.M., et al. (2022). Suppression of heterotopic ossification in fibrodysplasia ossificans progressiva using AAV gene delivery. Nat. Commun. *13*, 6175. https://doi.org/10.1038/s41467-022-33956-9.

24. Carlson, J.A., Rogers, B.B., Sifers, R.N., Finegold, M.J., Clift, S.M., DeMayo, F.J., Bullock, D.W., and Woo, S.L. (1989). Accumulation of PiZ alpha 1-antitrypsin causes liver damage in transgenic mice. J. Clin. Invest. *83*, 1183–1190. https://doi.org/10.1172/JCI113999.

25. Sinnett, S.E., Boyle, E., Lyons, C., and Gray, S.J. (2021). Engineered microRNA-based regulatory element permits safe high-dose miniMECP2 gene therapy in Rett mice. Brain *144*, 3005–3019. https://doi.org/10.1093/brain/awab182.

26. Borel, F., Gernoux, G., Cardozo, B., Metterville, J.P., Toro Cabrera, G.C., Song, L., Su, Q., Gao, G.P., Elmallah, M.K., Brown, R.H., and Mueller, C. (2016). Therapeutic rAAVrh10 Mediated *SOD1* Silencing in Adult *SOD1* ^{G93A} Mice and Nonhuman Primates. Hum. Gene Ther. *27*, 19–31. https://doi.org/10.1089/hum.2015.122.

27. Ling, C., Lu, Y., Kalsi, J.K., Jayandharan, G.R., Li, B., Ma, W., Cheng, B., Gee, S.W.Y., McGoogan, K.E., Govindasamy, L., et al. (2010). Human Hepatocyte Growth Factor Receptor Is a Cellular Coreceptor for Adeno-Associated Virus Serotype 3. Hum. Gene Ther. *21*, 1741–1747. https://doi.org/10.1089/hum.2010.075.

28. Miller, L.A., Royer, C.M., Pinkerton, K.E., and Schelegle, E.S. (2017). Nonhuman Primate Models of Respiratory Disease: Past, Present, and Future. ILAR J. *58*, 269–280. https://doi.org/10.1093/ilar/ilx030.

29. Keiser, M.S., Ranum, P.T., Yrigollen, C.M., Carrell, E.M., Smith, G.R., Muehlmatt, A.L., Chen, Y.H., Stein, J.M., Wolf, R.L., Radaelli, E., et al. (2021). Toxicity after AAV delivery of RNAi expression constructs into nonhuman primate brain. Nat. Med. *27*, 1982–1989. https://doi.org/10.1038/s41591-021-01522-3.

30. Rana, J., Marsic, D., Zou, C., Muñoz-Melero, M., Li, X., Kondratov, O., Li, N., De Jong, Y.P., Zolotukhin, S., and Biswas, M. (2023). Characterization of a Bioengineered AAV3B Capsid Variant with Enhanced Hepatocyte Tropism and Immune Evasion. Hum. Gene Ther. *34*, 289–302. https://doi.org/10.1089/hum.2022.176.

31. Hamilton, B.A., and Wright, J.F. (2021). Challenges Posed by Immune Responses to AAV Vectors: Addressing Root Causes. Front. Immunol. *12*, 675897. https://doi.org/10.3389/fimmu.2021.675897.

32. Crystal, R.G. (1989). The α1-antitrypsin gene and its deficiency states. Trends Genet. *5*, 411–417. https://doi.org/10.1016/0168-9525(89)90200-X.

33. Wright, J.F. (2020). Quantification of CpG Motifs in rAAV Genomes: Avoiding the Toll. Mol. Ther. *28*, 1756–1758. https://doi.org/10.1016/j.ymthe.2020.07.006.

34. Mendell, J.R., Al-Zaidy, S., Shell, R., Arnold, W.D., Rodino-Klapac, L.R., Prior, T.W., Lowes, L., Alfano, L., Berry, K., Church, K., et al. (2017). Single-Dose Gene-Replacement Therapy for Spinal Muscular Atrophy. N. Engl. J. Med. *377*, 1713–1722. https://doi.org/10.1056/NEJMoa1706198.

35. Mendell, Z.S., Kelly, L., Lowes, L., Reash, N., Iammarino, M., Alfano, L., Lewis, S., Church, K., Shell, R., Potter, R., et al. (2023). Long-term Safety and Efficacy in Patients with DMD 4 Years Post-Treatment with Delandistrogene Moxeparvovec (SRP-9001) in a Phase 1/2a Study (P3-8.006). Neurology *100*, 3700. https://doi.org/10.1212/WNL.0000000000203462.

36. Stegmeier, F., Hu, G., Rickles, R.J., Hannon, G.J., and Elledge, S.J. (2005). A lentiviral microRNA-based system for single-copy polymerase II-regulated RNA interference in mammalian cells. Proc. Natl. Acad. Sci. USA *102*, 13212–13217. https://doi.org/10.1073/pnas.0506306102.

37. Morgan, K., Marsters, P., Morley, S., Van Gent, D., Hejazi, A., Backx, M., Thorpe, E.R.K., and Kalsheker, N. (2002). Oncostatin M induced α1-antitrypsin (AAT) gene expression in Hep G2 cells is mediated by a 3′ enhancer. Biochem. J. *365*, 555–560. https://doi.org/10.1042/bj20011312.

38. Borel, F., Gernoux, G., Sun, H., Stock, R., Blackwood, M., Brown, R.H., and Mueller, C. (2018). Safe and effective superoxide dismutase 1 silencing using artificial microRNA in macaques. Sci. Transl. Med. *10*, eaau6414. https://doi.org/10.1126/scitranslmed.aau6414.

39. Sands, M.S. (2012). AAV-Mediated Liver-Directed Gene Therapy. In Adeno-Associated Virus Methods in Molecular Biology, R.O. Snyder and P. Moullier, eds. (Humana Press), pp. 141–157. https://doi.org/10.1007/978-1-61779-370-7_6.

40. Nathwani, A.C., Gray, J.T., Ng, C.Y.C., Zhou, J., Spence, Y., Waddington, S.N., Tuddenham, E.G.D., Kemball-Cook, G., McIntosh, J., Boon-Spijker, M., et al. (2006). Self-complementary adeno-associated virus vectors containing a novel liver-specific human factor IX expression cassette enable highly efficient transduction of murine and nonhuman primate liver. Blood *107*, 2653–2661. https://doi.org/10.1182/blood-2005-10-4035.

41. Sarkar, R., Tetreault, R., Gao, G., Wang, L., Bell, P., Chandler, R., Wilson, J.M., and Kazazian, H.H., Jr. (2004). Total correction of hemophilia A mice with canine FVIII using an AAV 8 serotype. Blood *103*, 1253–1260. https://doi.org/10.1182/blood-2003-08-2954.

42. Kattenhorn, L.M., Tipper, C.H., Stoica, L., Geraghty, D.S., Wright, T.L., Clark, K.R., and Wadsworth, S.C. (2016). Adeno-Associated Virus Gene Therapy for Liver Disease. Hum. Gene Ther. *27*, 947–961. https://doi.org/10.1089/hum.2016.160.

43. Maestro, S., Weber, N.D., Zabaleta, N., Aldabe, R., and Gonzalez-Aseguinolaza, G. (2021). Novel vectors and approaches for gene therapy in liver diseases. JHEP Rep. *3*, 100300. https://doi.org/10.1016/j.jhepr.2021.100300.

44. Domenger, C., and Grimm, D. (2019). Next-generation AAV vectors—do not judge a virus (only) by its cover. Hum. Mol. Genet. *28*, R3–R14. https://doi.org/10.1093/hmg/ddz148.

45. Liu, P., Liang, S.-Q., Zheng, C., Mintzer, E., Zhao, Y.G., Ponnienselvan, K., Mir, A., Sontheimer, E.J., Gao, G., Flotte, T.R., et al. (2021). Improved prime editors enable pathogenic allele correction and cancer modelling in adult mice. Nat. Commun. *12*, 2121. https://doi.org/10.1038/s41467-021-22295-w.

46. Packer, M.S., Chowdhary, V., Lung, G., Cheng, L.-I., Aratyn-Schaus, Y., Leboeuf, D., Smith, S., Shah, A., Chen, D., Zieger, M., et al. (2022). Evaluation of cytosine base editing and adenine base editing as a potential treatment for alpha-1 antitrypsin deficiency. Mol. Ther. *30*, 1396–1406. https://doi.org/10.1016/j.ymthe.2022.01.040.

OMTM, Volume *32*

Supplemental information

Biodistribution and safety of a single rAAV3B-AAT vector for silencing and replacement of alpha-1 antitrypsin in *Cynomolgus macaques*

Meghan Blackwood, Alisha M. Gruntman, Qiushi Tang, Debora Pires-Ferreira, Darcy Reil, Oleksandr Kondratov, Damien Marsic, Sergei Zolotukhin, Gwladys Gernoux, Allison M. Keeler, Christian Mueller, and Terence R. Flotte

Supplemental Figures

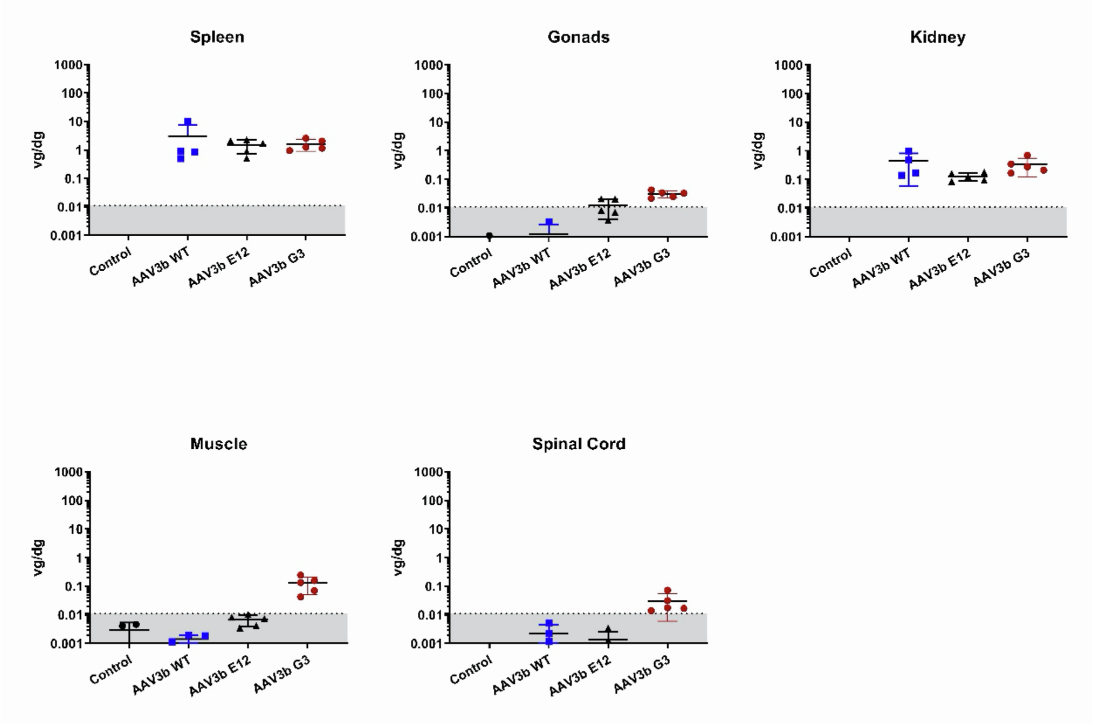

Figure S1 Biodistribution of the rAAV genome in several tissues showed no significant differences among the groups.

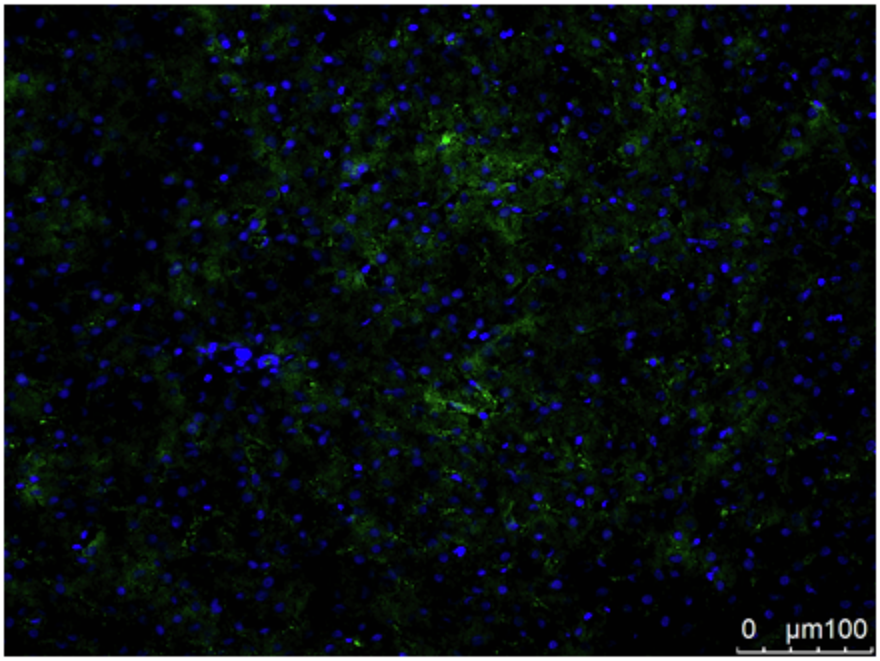

Figure S2 c-MYC staining of liver tissue from uninjected NHP showing minimal background fluorescence. Green: c-MYC; blue: DAPI.

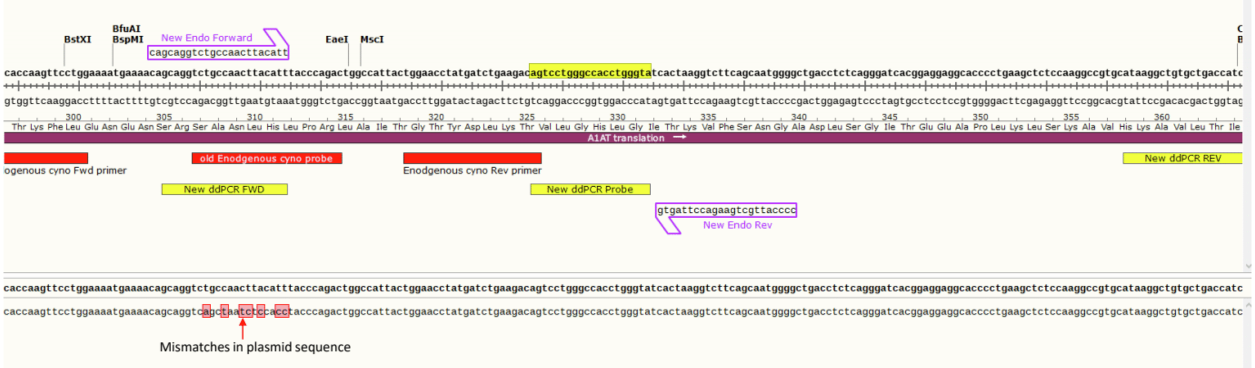

Figure S3 Design of primers for quantifying endogenous AAT gene levels and silent mutations in the gene intended to cause mismatches with the miRNA.

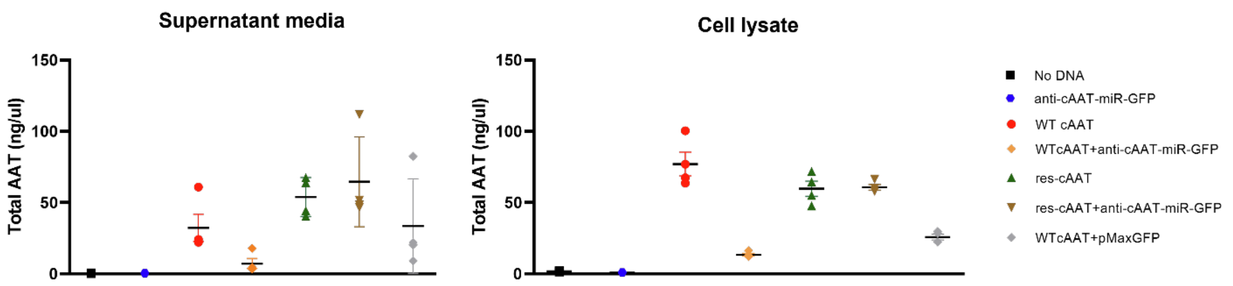

Figure S4 We designed experiments to confirm that constructs based on the cynomolgus alpha-1 antitrypsin (cAAT) sequence were effective for expression of wild-type cAAT (WT cAAT), silencing cAAT (anti-cAAT-miR) and expressing an allele of cAAT that is resist to that silencing miRNA (rec-cAAT). pMaxGFP served as a transfection control. Expression from the WT-cAAT cassette was effectively silenced by anti-cAAT miR, while the res-cAAT-expressing construct was not silenced.

Table S1 NHP groups in this study.

<table>
<tr><th></th><th>NHP ID</th><th>Capsid</th><th>Administration Route</th><th>Dose</th><th>Volume Rate</th></tr>
<tr><td rowspan="4">Group 1</td><td>1001 Male</td><td rowspan="4">AAV3B WT</td><td rowspan="4">IV</td><td rowspan="4">2.5E13 vg/kg
5E13 vg total</td><td rowspan="4">1 ml/min</td></tr>
<tr><td>1501 Female</td></tr>
<tr><td>1502 Female</td></tr>
<tr><td>1503 Female</td></tr>
<tr><td rowspan="5">Group 2</td><td>2001 Male</td><td rowspan="5">AAV3B-E13</td><td rowspan="5">IV</td><td rowspan="5">2.5E13 vg/kg
5E13 vg total</td><td rowspan="5">1 ml/min</td></tr>
<tr><td>2002 Male</td></tr>
<tr><td>2501 Female</td></tr>
<tr><td>2502 Female</td></tr>
<tr><td>2503 Female</td></tr>
<tr><td rowspan="5">Group 3</td><td>3001 Male</td><td rowspan="5">AAV3B-G3</td><td rowspan="5">IV</td><td rowspan="5">2.5E13 vg/kg
5E13 vg total</td><td rowspan="5">1 ml/min</td></tr>
<tr><td>3002 Male</td></tr>
<tr><td>3501 Female</td></tr>
<tr><td>3502 Female</td></tr>
<tr><td>3503 Female</td></tr>
</table>

Table S2. Neutralizing antibodies to AAV3B capsid prior and post dosing. NHP were prescreened for NAbs to the AAV vector before dosing. Only animals with NAb titers below 1:10 were selected. All the animals developed a neutralizing antibody response to AAV3B capsid post dosing (from Day 15 to Day 84).

Vector	Animal ID	Screening	Day 1 (Prior to dose)	Day 15	Day 57	Day 85
AAV3b-WT	1001	1:5	<1:5	1:10240	1:10240	1:2560
	1501	1:5	1:5	1:320	1:160	1:640
	1502	1:5	<1:5	1:80	<1:80	1:80
	1503	1:5	1:10	1:10240	1:160	1:80
AAV3b-E12	2001	1:5	<1:5	>1:20480	>1:20480	1:10240
	2002	1:5	<1:5	1:320	1:1280	1:320
	2501	1:5	1:10	1:320	1:160	1:320
	2502	1:5	<1:5	1:320	1:320	1:160
	2503	1:5	<1:5	1:320	1:160	1:320

**AAV3b-G3**	**3001**	**1:5**	**<1:5**	**1:320**	**1:1280**	**1:1280**
	**3002**	**1:5**	**<1:5**	**1:320**	**1:320**	**1:320**
	**3501**	**<1:5**	**<1:5**	**1:320**	**1:640**	**1:640**
	**3502**	**1:5**	**<1:5**	**1:640**	**1:320**	**1:320**
	**3503**	**1:5**	**<1:5**	**1:160**	**1:320**	**1:160**
